# Differential expression of genes in the RhoA/ROCK pathway in the hippocampus and cortex following intermittent hypoxia and high-intensity interval training

**DOI:** 10.1152/jn.00422.2023

**Published:** 2024-07-10

**Authors:** Natalie E. Doody, Nicole J. Smith, Elizabeth C. Akam, Graham N. Askew, Jessica C. F. Kwok, Ronaldo M. Ichiyama

**Affiliations:** ^1^School of Biomedical Sciences, University of Leeds, Leeds, United Kingdom; ^2^School of Psychology and Neuroscience, University of St Andrews, St Andrews, United Kingdom; ^3^Department of Cell and Molecular Biology, Karolinska Institute, Stockholm, Sweden; ^4^School of Sport, Exercise, and Health Sciences, Loughborough University, Loughborough, United Kingdom; ^5^Institute of Experimental Medicine, The Czech Academy of Sciences, Prague 4, Czech Republic

**Keywords:** exercise, inhibitory molecules, intermittent hypoxia, neuroplasticity, treadmill training

## Abstract

Structural neuroplasticity such as neurite extension and dendritic spine dynamics is enhanced by brain-derived neurotrophic factor (BDNF) and impaired by types of inhibitory molecules that induce growth cone collapse and actin depolymerization, for example, myelin-associated inhibitors, chondroitin sulfate proteoglycans, and negative guidance molecules. These inhibitory molecules can activate RhoA/rho-associated coiled-coil containing protein kinase (ROCK) signaling (known to restrict structural plasticity). Intermittent hypoxia (IH) and high-intensity interval training (HIIT) are known to upregulate BDNF that is associated with improvements in learning and memory and greater functional recovery following neural insults. We investigated whether the RhoA/ROCK signaling pathway is also modulated by IH and HIIT in the hippocampus, cortex, and lumbar spinal cord of male Wistar rats. The gene expression of 25 RhoA/ROCK signaling pathway components was determined following IH, HIIT, or IH combined with HIIT (30 min/day, 5 days/wk, 6 wk). IH included 10 3-min bouts that alternated between hypoxia (15% O_2_) and normoxia. HIIT included 10 3-min bouts alternating between treadmill speeds of 50 cm·s^−1^ and 15 cm·s^−1^. In the hippocampus, IH and HIIT significantly downregulated *Acan* and *NgR2* mRNA that are involved in the inhibition of neuroplasticity. However, IH and IH + HIIT significantly upregulated *Lingo-1* and *NgR3* in the cortex. This is the first time IH and HIIT have been linked to the modulation of plasticity-inhibiting pathways. These results provide a fundamental step toward elucidating the interplay between the neurotrophic and inhibitory mechanisms involved in experience-driven neural plasticity that will aid in optimizing physiological interventions for the treatment of cognitive decline or neurorehabilitation.

**NEW & NOTEWORTHY** Intermittent hypoxia (IH) and high-intensity interval training (HIIT) enhance neuroplasticity and upregulate neurotrophic factors in the central nervous system (CNS). We provide evidence that IH and IH + HIIT also have the capacity to regulate genes involved in the RhoA/ROCK signaling pathway that is known to restrict structural plasticity in the CNS. This provides a new mechanistic insight into how these interventions may enhance hippocampal-related plasticity and facilitate learning, memory, and neuroregeneration.

## Graphical abstract

**Figure:**
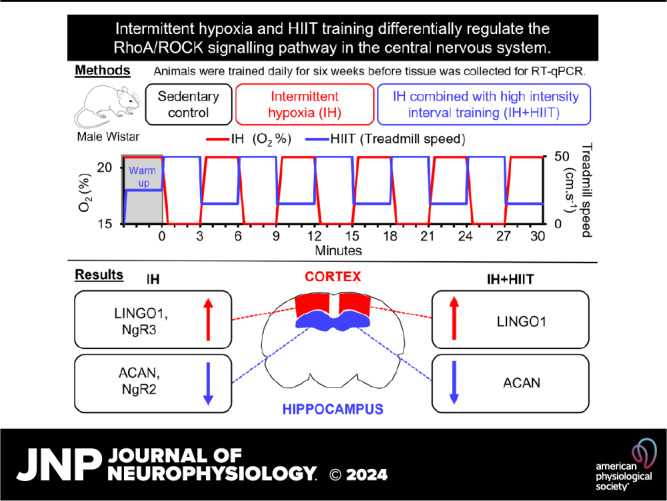

## INTRODUCTION

Structural neuroplasticity, such as neurite outgrowth and the regulation of dendritic spine dynamics, is required for learning and memory, and for the central nervous system (CNS) to recover from traumatic injuries and adapt to new experiences (1, 2). It is possible to enhance neuroplasticity using physiological interventions such as exposure to hypoxia and exercise training. Studies that expose rodents to intermittent hypoxia (IH) have shown an increase in neurogenesis in wild-type rodent models (3–5). Furthermore, IH has been documented to upregulate hippocampal brain-derived neurotrophic factor (BDNF) expression (6, 7), and rescue impairments of neurogenesis (6–8) and memory in models of stroke and Alzheimer’s pathology (6–9). Similarly, high-intensity interval training (HIIT) has been reported to enhance neurogenesis and memory (10, 11), and increase the hippocampal expression of BDNF (11–15).

BDNF is associated with neurogenesis, enhanced neurite extension, and the regulation of dendritic spine dynamics (16–19). There are also molecules within the CNS that contribute to growth cone collapse, limit neurite outgrowth, negatively regulate the formation of dendritic spines, and prevent synaptic reorganization (20–23), which we will refer to as “inhibitory molecules” or “inhibitory modulators of structural neuroplasticity” (outlined in more detail in the next paragraph). These inhibitory molecules are mainly known for impeding regeneration in the injured CNS (24) and for their role in closing the critical period during postnatal development (25–27). However, inhibitory molecules also limit structural neuroplasticity and regulate cytoskeletal/anatomical rearrangements in the healthy adult CNS (24). It is known that IH and HIIT upregulate BDNF, however, whether IH and HIIT also have the capacity to regulate factors that restrict structural neuroplasticity has currently been overlooked. It is possible that IH and HIIT may simultaneously “take a foot off the inhibitory brakes” and facilitate the “acceleration” of BDNF to create a more permissive environment for structural neuroplasticity, although this is yet to be investigated.

Within this paper, we refer to three subtypes of transmembrane or extracellular inhibitory modulators of neuroplasticity: myelin-associated inhibitors, repulsive guidance molecules, and chondroitin sulfate proteoglycans (CSPGs) (28). The myelin-associated inhibitors, Nogo-A, myelin-associated glycoprotein (MAG), and oligodendrocyte myelin glycoprotein (OMGp) are known to inhibit neurite outgrowth and are expressed in oligodendrocytes and some neurons (29). Oligodendrocytes also express repulsive guidance molecules such as semaphorin-4D (SEMA4D) and ephrin-B3 (EFNB3) that can induce growth cone collapse and restrict axonal outgrowth (30, 31). Finally, CSPGs include a family of lecticans (aggrecan, brevican, neurocan, and versican) that are expressed in the extracellular matrix and are integral components of structures called perineuronal nets (PNNs) that stabilize synapses and restrict plasticity (23). The inhibitory modulators of plasticity are ligands in the RhoA/rho-associated coiled-coil containing protein kinase (ROCK) signaling pathway that regulates dendritic spine morphology, actin cytoskeleton dynamics, growth cone collapse, and therefore the inhibition of neurite outgrowth (29, 32). The inhibitory ligands bind to their respective membrane receptors that converge to activate the RhoA/ROCK signaling pathway. The membrane receptors include Nogo receptors (NgR1, NgR2, NgR3), a NgR1/LINGO-1/TROY receptor complex, the semaphorin and ephrin receptors PLEXIN-B1 and EPHA4, and CSPG receptors (NgR1, NgR3, PTPRF, PTPRS) (33–36). Once the ligand-receptor complex is formed, guanine exchange factors (GEFs) are activated that convert guanosine diphosphate (GDP) to guanosine triphosphate (GTP), causing the activation of the Rho small GTPase, RhoA (29). In addition, the Nogo-Δ20 component of Nogo-A activates G protein 13 (G13) through the Sphingosine-1-phosphate receptor 2 (S1PR2) receptor, followed by the activation of rho guanine nucleotide exchange factor 12 (LARG), which leads to RhoA GTP activation (29, 37). SEMA4D/PLEXIN-B1 signaling can also mediate the LARG-induced activation of RhoA GTP (38). When RhoA is in its GTP-bound form, it activates rho-associated coiled-coil containing protein kinase (ROCK) that triggers a cascade of downstream signaling. Two isoforms of ROCK exist, with ROCK 2 being the dominant isoform in the CNS, and cardiac and skeletal muscle, whereas ROCK 1 is the dominant isoform in the lungs, liver, blood, and immune system (39). The effectors downstream of ROCK 2 include LIM domain kinase 1 (LIMK1), myosin light chain 2 (MYL2), phosphatase and tensin homolog (PTEN), collapsin response mediator protein 2 (CRMP2), and cofilin 1 (CFL1), which regulate growth cone collapse, neurite outgrowth inhibition, actin depolymerization, and dendritic spine dynamics (35, 40, 41).

Voluntary wheel running has been documented to reduce levels of Nogo-A and MAG in the hippocampus (42), Nogo-A in the cortex (43), and MAG in the spinal cord (44). Furthermore, CSPGs were upregulated in the lumbar spinal cord yet downregulated in the hippocampus following voluntary wheel running (45). These results show that exercise has the capacity to regulate levels of RhoA/ROCK pathway inhibitory ligands, therefore, it is plausible that exercise training may modulate other components of the signaling pathway.

As both IH and HIIT have previously been shown to enhance BDNF in the CNS, we hypothesized that these interventions would also reduce the expression of the opposing inhibitory molecules. Due to the common convergence of inhibitory molecules at RhoA/ROCK signaling, we decided to investigate how components of the RhoA/ROCK pathway respond to IH and HIIT in the CNS of healthy adults. Twenty-five genes from the RhoA/ROCK pathway were selected, and their expressions upon IH, HIIT, or IH + HIIT were investigated in the hippocampus, cortex, and lumbar spinal cord in adult male rats. The target genes included inhibitory ligands, their respective membrane receptors, and downstream signaling molecules involved in RhoA/ROCK signaling to identify which part of the pathway was modulated by IH, HIIT, or IH + HIIT. The results provide a fundamental step toward elucidating the interaction between neurotrophic factors (BDNF) and inhibitory molecules and how they regulate structural neuroplasticity in response to physiological interventions. This may in the future be manipulated to benefit conditions in which neurite extension and dendritic spine formation are required, e.g., improving cognition, delay age-related memory loss, and neurorehabilitation.

## MATERIALS AND METHODS

### Animals

Adult male Wistar Han rats (8 wk old, ∼200 g) were purchased from Charles River Laboratories (Canterbury, UK, *n* = 16). The majority of literature that informed the rationale and design for this study was conducted in males (4–10, 12–15, 42, 43, 46–48) opposed to females (11, 45), therefore male rats were used in this study to remain in accordance with the majority of the current literature. Animals were housed four per cage with ad libitum access to food and water, at 20 ± 1°C, and under a 12-h light/dark cycle at Central Biomedical Services (University of Leeds, UK). All procedures were conducted during the light cycle, complied with the UK Animals (Scientific Procedures) Act 1986 (ASPA), and were approved by the University of Leeds Animal Welfare and Ethical Review Committee (PPL 70/8085).

### Study Overview

Animals were familiarized on a treadmill for five days before being allocated into three experimental groups: cage control (CON) (*n* = 4); intermittent hypoxia (IH) (*n* = 4); high-intensity interval training (HIIT) (*n* = 4), and intermittent hypoxia combined with high-intensity interval training (IH + HIIT) (*n* = 4). The rats were subjected to their experimental conditions five days a week for 6 wk. After 6 wk, animals were anesthetized using sodium pentobarbital and decapitated to collect fresh CNS tissue for analyzing gene expression of RhoA/ROCK pathway components.

### Treadmill Familiarization

Animals were familiarized on a motorized treadmill (Panlab, Harvard apparatus, Cambourne, UK) 5 min a day for five days. The treadmill speed was gradually increased from 0 cm·s^−1^ to 50 cm·s^−1^ across the five days (Table 1). If animals faced toward the back of the treadmill the speed was lowered to enable them to return to forward facing and then raised again.

**Table 1.** Treadmill familiarization speeds

Minutes	*Day 1*	*Day 2*	*Day 3*	*Day 4*	*Day 5*
0–1	0 cm·s^−1^	20 cm·s^−1^	25 cm·s^−1^	25 cm·s^−1^	25 cm·s^−1^
1–2	0 cm·s^−1^	25 cm·s^−1^	30 cm·s^−1^	30 cm·s^−1^	30 cm·s^−1^
2–3	20 cm·s^−1^	30 cm·s^−1^	35 cm·s^−1^	35 cm·s^−1^	35 cm·s^−1^
3–4	20 cm·s^−1^	35 cm·s^−1^	40 cm·s^−1^	40 cm·s^−1^	40 cm·s^−1^
4–4:30	25 cm·s^−1^	40 cm·s^−1^	45 cm·s^−1^	45 cm·s^−1^	45 cm·s^−1^
4:30–5	25 cm·s^−1^	40 cm·s^−1^	50 cm·s^−1^	50 cm·s^−1^	50 cm·s^−1^

### Experimental Group Allocation

To avoid bias in the exercise compliance of experimental groups, the animals were given a performance rating for each day during familiarization: good runner (maintained continuous running)—two points; average runner (some turning/hanging back)—one point; and bad runner (mostly refusing to run)—zero points. Each animal was given a total score out of 10 to measure familiarization compliance, and ranked by performance (1 the best, 16 the worst). The performance rankings were divided into quartiles. One animal from each quartile was allocated to each experimental group (*n* = 4). It is important to note that animals turning on the treadmill were observed during familiarization when treadmill speeds were low. All animals learned/adhered to the exercise protocol.

### Experimental Conditions

#### Intermittent hypoxia.

During IH, animals were placed in the lane of the treadmill chamber in which the level of hypoxia could be manipulated. The IH protocol ran for a total of 30 min, 5 days a week, for 6 wk. Five 3-min bouts of hypoxia (14.99% O_2_ ± 0.19) were alternated with five 3-min bouts of normoxia. Throughout hypoxic treatment O_2_% in the treadmill chamber was continuously monitored. To achieve the desired level of hypoxia (15% O_2_), N_2_ was flowed in at ∼4.5 L·min^−1^ and air was extracted from the treadmill chamber at 17.6 L·min^−1^ using a vacuum pump (Model DOA-P504-BN, GAST Manufacturing Inc., MI), controlled by a mass flow controller (MFC-2, Sable Systems International Inc., NV), and a mass flow control valve (840 Side-Trak Mass Flow Controller; Sierra Instruments Inc., CA). Air was subsampled at ∼200 mL·min^−1^ and the difference in O_2_ content between chamber air and outside air was determined using a custom-made differential O_2_ analyzer based on a zirconia sensor (Zr703, Servomex, Crowborough, UK). The outputs from the gas analyzer were recorded via a PowerLab using LabChart software (ADInstruments Inc., CO). The analyzer was calibrated before each animal using a specialist gas mixture of 15% O_2_ balanced with N_2_ (Certified value: 15.18%, BOC Gases, Leeds, UK) and outside air scrubbed of CO_2_ (20.95% O_2_ and 0% CO_2_).

#### Response times.

From the onset of N_2_ being flowed into the chamber at the beginning of the hypoxic interval, it took ∼28 s for the oxygen concentration to drop to 90% of the desired hypoxia level. When the full level of desired hypoxia was achieved, the chamber remained at a steady state for the rest of the interval. At the end of the hypoxic interval, the flow of N_2_ into the chamber ceased, and it took ∼25 s for the chamber oxygen concentration to return to 90% of baseline levels. These response times were calculated while animals were in the chamber being exposed to hypoxia.

#### High-intensity interval training.

HIIT animals ran for a total of 33 min on the treadmill at 5° inclination, 5 days a week, for 6 wk. The HIIT protocol consisted of a 3-min warm up at 25 cm·s^−1^, followed by five cycles of: 3 min of running at 50 cm·s^−1^, and 3 min walking at 15 cm·s^−1^. The highest speed of 50 cm·s^−1^ was selected as this was the maximum speed that the rats were willing to run. The high-intensity interval speed (50 cm·s^−1^) corresponds to ∼86% V̇o_2max_ in 8-wk-old male Wistar rats (49).

#### Intermittent hypoxia and high-intensity interval training.

IH + HIIT animals performed the HIIT protocol while been exposed to IH. The warm-up was performed in normoxia. In an attempt to maximize the neural response to the hypoxic and exercise stimuli, the bouts were synchronized so that rats were exercising at the higher intensity during the lower oxygen interval.

#### Cage control.

Cage control animals remained sedentary and were not exposed to hypoxic training throughout the experimental period.

### Tissue Collection

Animals were overdosed with an intraperitoneal injection of sodium pentobarbital (200 mg/kg) to induce deep anesthesia and the absence of blink and pedal reflexes. Following decapitation, the hippocampus, cortex (directly above the hippocampus), and lumbar region of the spinal cord were rapidly dissected, snap frozen in liquid nitrogen and stored at −80°C. Storage time was equivalent for all samples throughout the entire process.

### Gene Expression

#### Reverse transcription quantitative polymerase chain reaction.

Fresh CNS regions (hippocampus, cortex, and lumbar spinal cord) were separately homogenized for 10 min in 700 µL of TRIzol using 7-mm stainless steel ball bearings and a TissueLyser LT bead mill (Qiagen, Hilden, Germany). RNA was extracted using TRIzol and the PureLink RNA Mini Kit with On-column PureLink DNase Treatment (Thermo Fisher Scientific, Loughborough, UK). RNA extraction was performed in batches of eight samples. RNA concentration and purity were determined with a NanoDrop ND-2000 spectrophotometer. RNA was diluted to 200 ng·µL^−1^ using ultra-pure nuclease-free water.

cDNA was reverse transcribed from 2 µg of RNA using the Precision nanoScript TM2 Reverse Transcription kit (PrimerDesign, Hampshire, UK). cDNA was diluted to 5 ng·µL^−1^ using ultra-pure nuclease-free water. Custom TaqMan Array Plates were used to quantify the relative gene expression of 25 genes within the RhoA/ROCK signaling pathway of neurite outgrowth inhibition. The wells of the TaqMan Array Plates were pre-loaded with dried-down TaqMan Gene Expression Assays with PCR efficiencies of 100% (see Table 2 for gene assay list). The plates were briefly centrifuged before adding the cDNA and PCR master mix. Each 20 µL reaction contained 10 µL of cDNA at 2 ng/µL (total cDNA per reaction: 20 ng) and 10 µL of TaqMan Fast Advanced Master Mix (Thermo Fisher Scientific, Loughborough, UK). Plates were sealed with optical adhesive film, briefly centrifuged, and loaded on a CFX96 Touch Real-Time PCR Detection System (Bio-Rad, Watford, UK). The thermal protocol included a uracil-*N*-glycosylase (UNG) incubation at 50°C for 2 min, an enzyme activation period at 95°C for 20 s, and 40 cycles of a 1-s denaturing step at 95°C and a 20 s anneal/extension step at 60°C. Each sample was run in duplicate for no template control wells and for each gene expression assay. Gene expression experiments were informed by the MIQE guidelines (50).

**Table 2.** TaqMan gene expression assay information (RhoA/ROCK pathway)

Molecule Type	Gene Name	Gene Type	TaqMan Gene Symbol	TaqMan Assay ID	Amplicon Length
Reference genes	*18s*	Reference	18s RNA	Hs99999901_s1	
	*CyPA*	Reference	Ppia	Rn00690933_m1	
Inhibitory ligands	*Nogo-A*	Target	Rtn4	Rn00582903_m1	90
	*MAG*	Target	Mag	Rn01457782_m1	56
	*OMGp*	Target	Omg	Rn02533851_s1	131
	*SEMA4D*	Target	Sema4d	Rn01435039_m1	62
	*EFNB3*	Target	Efnb3	Rn01750591_g1	82
	*ACAN*	Target	Acan	Rn00573424_m1	74
	*VCAN*	Target	Vcan	Rn01493755_m1	89
	*NCAN*	Target	Ncan	Rn00581331_m1	59
	*BCAN*	Target	Bcan	Rn00563814_m1	71
Membrane receptors	*NgR1*	Target	Rtn4r	Rn00586061_s1	57
	*NgR2*	Target	Rtn4rl2	Rn00710574_m1	63
	*NgR3*	Target	Rtn4rl1	Rn01466695_m1	119
	*TROY*	Target	Tnfrsf19	Rn01534699_m1	60
	*LINGO-1*	Target	Lingo1	Rn03993618_s1	109
	*LPAR1*	Target	Lpar1	Rn00588435_m1	67
	*PTPRF*	Target	Ptprf	Rn00695914_m1	70
	*PTPRS*	Target	Ptprs	Rn00569511_m1	76
Intracellular signaling	*ROCK2*	Target	Rock2	Rn00564633_m1	73
	*GNA13*	Target	Gna13	Rn01461471_m1	106
	*LARG*	Target	Arhgef12	Rn01417838_m1	101
	*LIMK1*	Target	Limk1	Rn01499352_m1	69
	*MYL2*	Target	Myl2	Rn01480558_g1	94
	*PTEN*	Target	Pten	Rn00477208_m1	73
	*CRMP2*	Target	Dpysl2	Rn01534654_m1	73
	*CFL1*	Target	Cfl1	Rn01501422_g1	77

ACAN, aggrecan; BCAN, brevican; CFL1, Cofilin 1; CRMP2, Collapsin response mediator protein 2; CyPA, cyclophilin A; EFNB3, ephrin-B3; GNA13, guanine nucleotide-binding protein, alpha 13; LARG, rho guanine nucleotide exchange factor 12; LIMK1, LIM domain kinase 1; LINGO-1, Leucine rich repeat and Ig domain containing 1; LPAR1, lysophosphatidic acid receptor 1; MAG, myelin-associated glycoprotein; MYL2, myosin light chain 2; NCAN, neurocan; NgR1, Nogo receptor 1; NgR2, Nogo receptor 2; NgR3, Nogo receptor 3; OMGp, oligodendrocyte-myelin glycoprotein; PTEN, phosphatase and tensin homolog; PTPRF, protein tyrosine phosphatase, receptor type, F; PTPRS, Protein tyrosine phosphatase, receptor type, S; ROCK2, rho-associated coiled-coil containing protein kinase 2; SEMA4D, semaphorin-4D; TROY, tumor necrosis factor receptor superfamily, member 19; VCAN, versican.

#### Data processing.

Relative quantification of target genes was calculated using the 2^−ΔΔCT^ method. Relative gene expression was normalized by subtracting the geometric mean of the reference gene Cq values (reference genes: 18s and Cyclophilin A). Data were excluded if the Cq values were above cycle 35 or outside two standard deviations from the mean. Group sizes for each gene after exclusions were applied are presented in Supplemental Table S1. A Log_2_ transformation was applied to the 2^−ΔΔCT^ data before statistical testing was performed to identify statistically significant changes in gene expression. As all genes analyzed were part of the RhoA/ROCK pathway, smaller gene changes that were not deemed statistically significant individually may still be of biological relevance when observed as a full pathway. Thus, relative fold change cut-off values of ≤0.75 and ≥1.5 (from untransformed 2^−ΔΔCT^ values) were used to further explore the gene expression profiles of the RhoA/ROCK pathway (51–54). Figures were produced using GraphPad Prism version 10.1.0 for Mac (GraphPad Software, CA). Venn diagrams were created using InteractiVenn (55) (http://www.interactivenn.net/).

### Statistical Analysis

All statistical analysis was performed in IBM SPSS software version 26 (IBM). Shapiro–Wilk tests determined normality prior to conducting statistical tests. For normally distributed target genes, one-way ANOVAs followed by Bonferroni-corrected post hoc tests were used to determine significant differences in relative gene expression between experimental groups. For target genes that were not normally distributed, Kruskal–Wallis H tests followed by Dunn’s multiple comparison tests and the Bonferroni correction were used. All statistical analyses were performed on Log_2_ transformed data. G*Power software version 3.1.9.7 was used to compute effect sizes (Cohen’s *d*) for all statistically significant target genes. Data in figures were presented as means ± standard error of the mean (SE). The α level was set at 0.05 and statistical significance was denoted in figures with an asterisk (*P < 0.05).

## RESULTS

**Figure 1.**
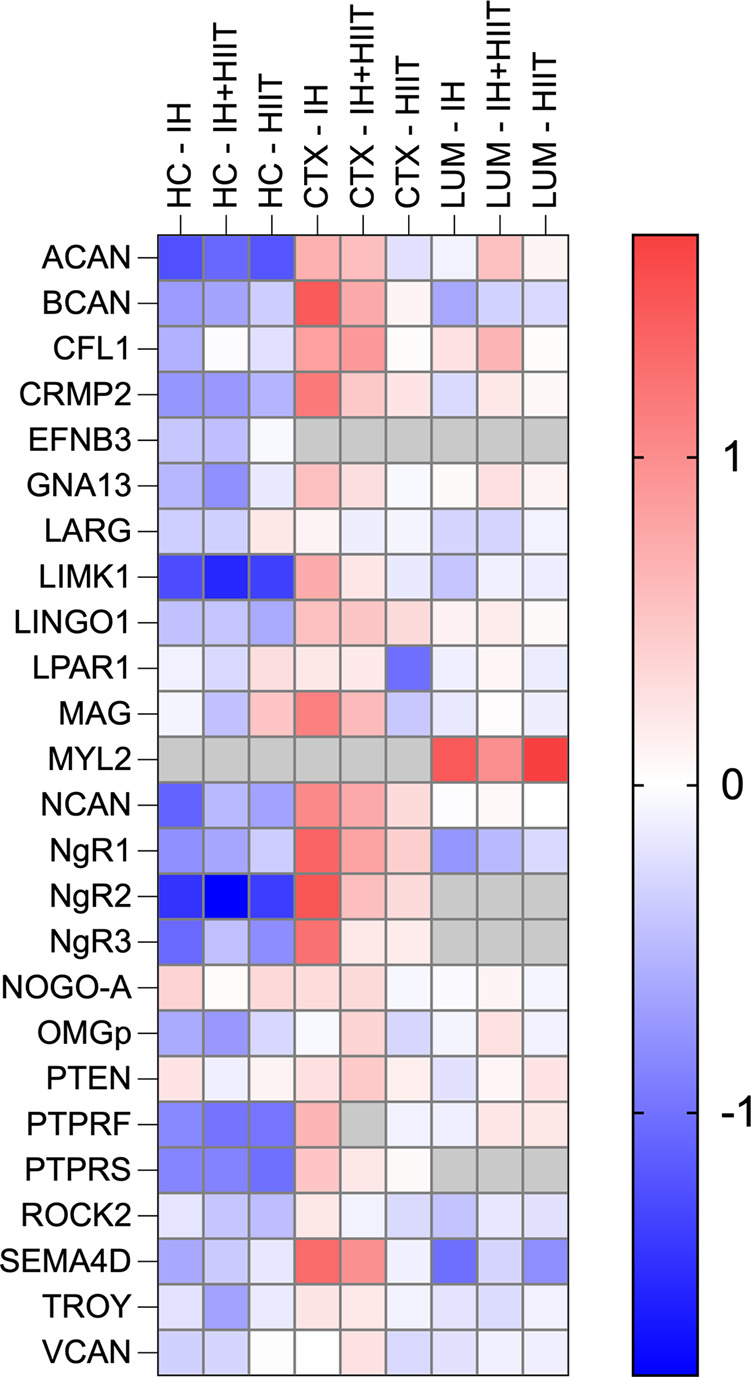
The effects of intermittent hypoxic (IH) training and intermittent hypoxia combined with high-intensity interval training (HIIT) on the transcription of RhoA/rho-associated coiled-coil containing protein kinase (ROCK) signaling pathway components in the central nervous system. Heatmap presenting the Log_2_ fold change (Log_2_ FC) of genes involved in the RhoA/ROCK signaling pathway following 6 wk of intermittent hypoxia (IH), intermittent hypoxia with high intensity interval training (IH + HIIT), or high-intensity interval training (HIIT) in adult male Wistar rats compared with sedentary animals (Log_2_ FC 0). Gene expression profiles were determined using the 2^−ΔΔCT^ method for the following regions: hippocampus (HC—first three columns); cortex (CTX—middle three columns); and lumbar spinal cord (LUM—last three columns). Blue—downregulated mRNA expression, red—upregulated mRNA expression, gray—no remaining data point after exclusion criteria applied.

To investigate whether inhibitory molecules in the CNS were modulated by IH, HIIT, and IH + HIIT, RT-qPCR was used to determine the gene expression of 25 RhoA/ROCK signaling pathway components including inhibitory ligands, membrane receptors, and downstream signaling molecules (Table 2). Gene expression profiles of the RhoA/ROCK pathway following 6 wk of IH, IH + HIIT, and HIIT (30 min a day, 5 days a week) are displayed in Fig. 1. In general, IH, IH + HIIT, and HIIT reduced the expression of multiple genes that are involved in the inhibition of neurite outgrowth within the hippocampus. Conversely, IH and IH + HIIT increased the expression of RhoA/ROCK pathway components in the cortex.

### IH, IH ± HIIT, and HIIT Downregulated RhoA/ROCK Pathway Genes in the Hippocampus

**Figure 2.**
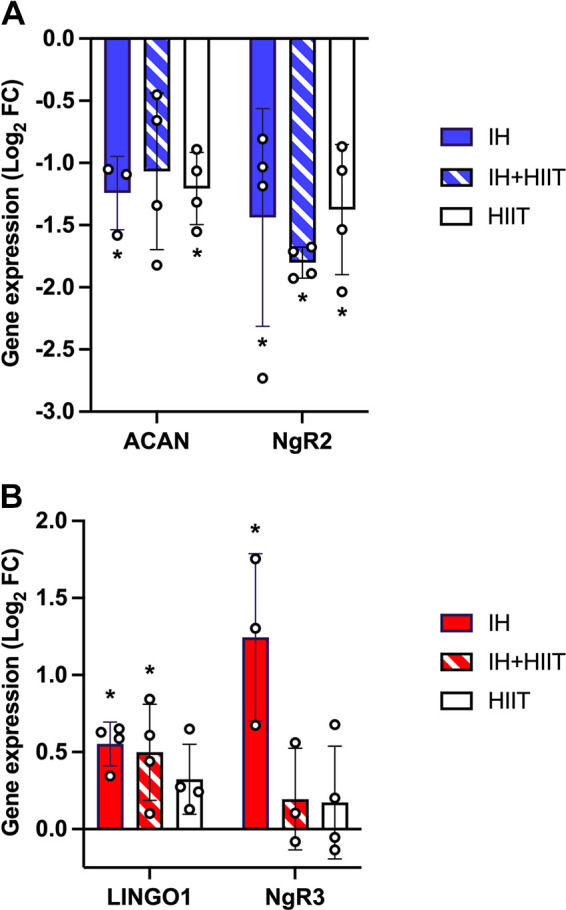
Novel candidates in the regulation of neuroplasticity following intermittent hypoxic training and intermittent exercise training. Changes in the expression of RhoA/rho-associated coiled-coil containing protein kinase (ROCK) pathway genes following 6 wk of daily intermittent hypoxia (IH), intermittent hypoxia with high-intensity interval training (IH + HIIT), high-intensity interval training (HIIT) in the hippocampus (*A*) and cortex (*B*) of adult male Wistar rats compared with sedentary animals. Data are presented as the mean Log_2_ fold change (Log_2_ FC) and error bars represent standard error of the mean. Depending on normality checks, differences in gene expression were assessed using one-way ANOVAs or Kruskal–Wallis *H* tests. Statistical significance determined by post hoc tests denoted with *P < 0.05 compared with sedentary control, *n* = 3 or 4. Blue—downregulated mRNA expression, red—upregulated mRNA expression.

Of the 25 RhoA/ROCK pathway genes analyzed, two genes showed transcriptional changes following IH, IH + HIIT, and HIIT in the hippocampus: *Acan and NgR2* (Fig. 2*A*). There was a statistically significant reduction in the expression of *Acan*, which encodes the CSPG aggrecan, following both IH and HIIT [*F*(3,10) = 5.958, *P* = 0.013, effect size: 0.85], [IH: −1.2415 ± 0.2949 Log_2_ fold change (FC), *P* = 0.031], (IH + HIIT: −1.0680 ± 0.6303 Log_2_ FC, *P* = 0.050), (HIIT: −1.2057 ± 0.2897 Log_2_ FC, *P* = 0.025). In addition, IH, IH + HIIT, and HIIT all downregulated the expression of *NgR2* (Nogo-receptor 2—a receptor for MAG) [*F*(3,12) = 7.671, *P* = 0.004, effect size: 0.79), (IH: −1.4389 ± 0.8749 Log_2_ FC, *P* = 0.024), (IH + HIIT: −1.8016 ± 0.1254 Log_2_ FC, *P* = 0.005), (HIIT: −1.3752 ± 0.5226 Log_2_ FC, *P* = 0.032). All hippocampal gene expression data and statistical analyses for RhoA/ROCK pathway components are presented in Supplemental Table S2.

### IH and IH ± HIIT Upregulated RhoA/ROCK Pathway Genes in the Cortex

In contrast to the downregulated hippocampal gene profile, intermittent hypoxic training upregulated two RhoA/ROCK pathway genes in the cortex: *Lingo1 and NgR3* (Fig. 2*B*). Both IH and IH + HIIT upregulated the mRNA expression of the membrane receptor, *Lingo-1* [*F*(3,12) = 5.382, *P* = 0.014, effect size: 0.70], (IH: 0.5527 ± 0.1415 Log_2_ FC, *P* = 0.020), (IH + HIIT: 0.4988 ± 0.3123 Log_2_ FC, *P* = 0.039), (HIIT: 0.3234 ± 0.2264 Log_2_ FC, *P* = 0.328). Nogo-receptor 3 (*NgR3*), a receptor for CSPGs (56), had a statistically significant upregulation in mRNA expression following IH compared with cage control animals [*F*(3,9) = 5.500, *P* = 0.020, effect size: 0.91], (IH: 1.2440 ± 0.5430 Log_2_ FC, *P* = 0.034), (IH+ HIIT: 0.1945 ± 0.3302 Log_2_ FC, *P* = 1.000), (HIIT: 0.1724 ± 0.3664 Log_2_ FC, *P* = 1.000). Kruskal–Wallis *H* tests identified *Ncan* and *Sema4d* to be significantly upregulated following IH and IH + HIIT [*Ncan: H*(3) = 8.625, *P* = 0.035, effect size: 0.65; *Sema4d: H*(3) = 10.787, *P* = 0.013, effect size: 0.82]. However, the pairwise comparisons did not remain statistically significant after the Bonferroni correction was applied [*Ncan* (IH: 1.0320 ± 0.6144 Log_2_ FC, *P* = 0.056), (IH + HIIT: 0.7540 ± 0.2374 Log_2_ FC, *P* = 0.128), (HIIT: 0.3104 ± 0.3815 Log_2_ FC, *P* = 1.000); *Sema4d* (IH: 1.2949 ± 0.7401 Log_2_ FC, *P* = 0.155), (IH + HIIT: 0.9708 ± 0.2928 Log_2_ FC *P* = 0.270), (HIIT: −0.1071 ± 0.3607 Log_2_ FC, *P* = 1.000)]. All cortical gene expression data and statistical analyses for RhoA/ROCK pathway components are presented in Supplemental Table S3.

### RhoA/ROCK Pathway Genes Show a Divergent Transcriptional Response to Hypoxia and Exercise in the Cortex

The overall gene expression profiles of the RhoA/ROCK pathway following IH, IH + HIIT, and HIIT showed a divergent response to hypoxia and exercise training in the cortex (Gene expression profiles presented in Fig. 1). Of 21 genes, 19 (90.5%) showed higher Log_2_ FC values than cage control animals in both the IH and IH + HIIT groups. However, when IH was combined with HIIT, the gene expression of 16 (76.2%) of these RhoA/ROCK pathway genes was reduced compared with IH alone. In addition, following HIIT the majority of RhoA/ROCK pathway genes (11 genes: 52.4%) showed lower Log_2_ FC values than cage control animals.

Although there were no statistically significant target genes when comparing HIIT to cage control animals, there were three genes in which mRNA expression was significantly lower in HIIT animals compared with animals that were exposed to hypoxia: *Lpar1*, *Mag*, and *Omgp*. The expression of *Lpar1*, a lysophosphatidic acid receptor that activates RhoA signaling (57), was shown to be lower in HIIT animals (HIIT: −1.0186 ± 0.5002 Log_2_ FC) compared with to both IH and IH + HIIT [*F*(3,12) = 5.382, *P* = 0.014, effect size: 0.85], (IH: 0.2030 ± 0.4927 Log_2_ FC, *P* = 0.029), (IH + HIIT: 0. 0.5940 Log_2_ FC, *P* = 0.032). In HIIT animals, the cortical expression of the myelin-associated inhibitors *Mag* and *Omgp* was also significantly lower compared with IH and IH + HIIT, respectively {*Mag* [*H*(3) = 8.801, *P* = 0.032, effect size: 0.64]}, (HIIT: −0.3917 ± 0.4684 Log_2_ FC), (IH: 1.1030 ± 0.8846 Log_2_ FC, *P* = 0.036); *Omgp* [*F*(3,12) = 3.920, *P* = 0.037, effect size: 0.65], (HIIT: −0.2937 ± 0.2554 Log_2_ FC), (IH + HIIT: 0.3641 ± 0.3565 Log_2_ FC, *P* = 0.032).

### IH Differentially Regulated RhoA/ROCK Pathway Genes in the Hippocampus and Cortex

**Figure 3.**
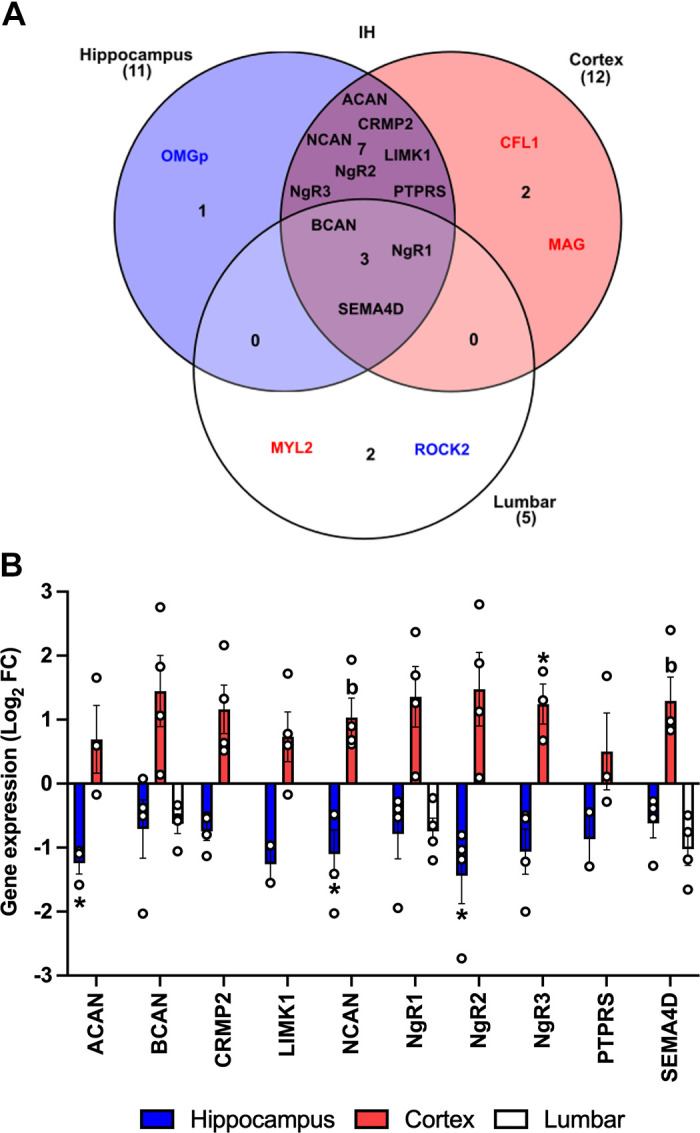
Differentially expressed RhoA/rho-associated coiled-coil containing protein kinase (ROCK) pathway genes following intermittent hypoxia (IH) in the central nervous system (CNS). *A*: gene expression profiles were explored using the untransformed 2^−ΔΔCT^ cut-off values of ≤0.75 and ≥1.5 for relative fold change (FC) data to identify differentially expressed genes (DEGs) in the hippocampus, cortex, and lumbar regions after 6 wk of intermittent hypoxia (IH). Blue text—downregulated genes; red text—upregulated genes; black text—DEGs that have been identified in more than one CNS region with divergent expression patterns. *B*: the expression profiles of DEGs after IH in the CNS. The relative fold change expression data for DEGs was Log_2_ transformed before statistical analysis. Blue and white bars—downregulated mRNA expression; red bars—upregulated mRNA expression. Depending on normality checks, differences in gene expression were assessed using one-way ANOVAs or Kruskal—Wallis *H* tests. Statistical significance determined by post hoc tests denoted with *P < 0.05 compared with sedentary control, ^b^*P* < 0.05 compared with sedentary control before adjustment (pairwise comparisons not statistically significant after the Bonferroni correction).

Throughout the CNS, only a small number of the 25 RhoA/ROCK pathway genes analyzed were reported to have a statistically significant difference in mRNA expression following IH: two genes in the hippocampus (*Acan* and *NgR2*) and two genes in the cortex (*Lingo-1* and *NgR3*). However, when looking at gene expression patterns for all target genes with available data following IH, in the hippocampus 19 of 21 genes (91%) had negative Log_2_ FC values (downregulated); in the cortex 18 of 20 genes (90%) had positive Log_2_ FC values (upregulated); and in the lumbar spinal cord 16 of 19 genes (84%) had negative Log_2_ FC values (downregulated), (Supplemental Tables S2, S3, and S4). As all the analyzed genes are involved in the same biological pathway, the accumulation of smaller nonstatistically significant gene changes may still have the potential to impact the function of the signaling pathway. Therefore, relative fold change cut-off values of ≤0.75 and ≥1.5 (51–54) were applied to untransformed 2^−ΔΔCT^ data to explore the gene expression profiles of the overall RhoA/ROCK pathway more comprehensively. All genes that were identified to be outside the cut off values are referred to as differentially expressed genes (DEGs) and are presented in Supplemental Table S5. Following IH, there were three DEGs that were identified in all three CNS regions: *Bcan, NgR1*, and *Sema4d* (Fig. 3*A*). These DEGs were downregulated in the hippocampus and lumbar spinal cord yet were upregulated in the cortex (Fig. 3*B*). In addition, seven DEGs showed the same divergent response of being downregulated in the hippocampus and upregulated in the cortex (*Acan, Crmp2, Limk1, Ncan, NgR2, NgR3,* and *Ptprs*).

### IH ± HIIT Differentially Regulated RhoA/ROCK Pathway Genes in the Hippocampus and Cortex

**Figure 4.**
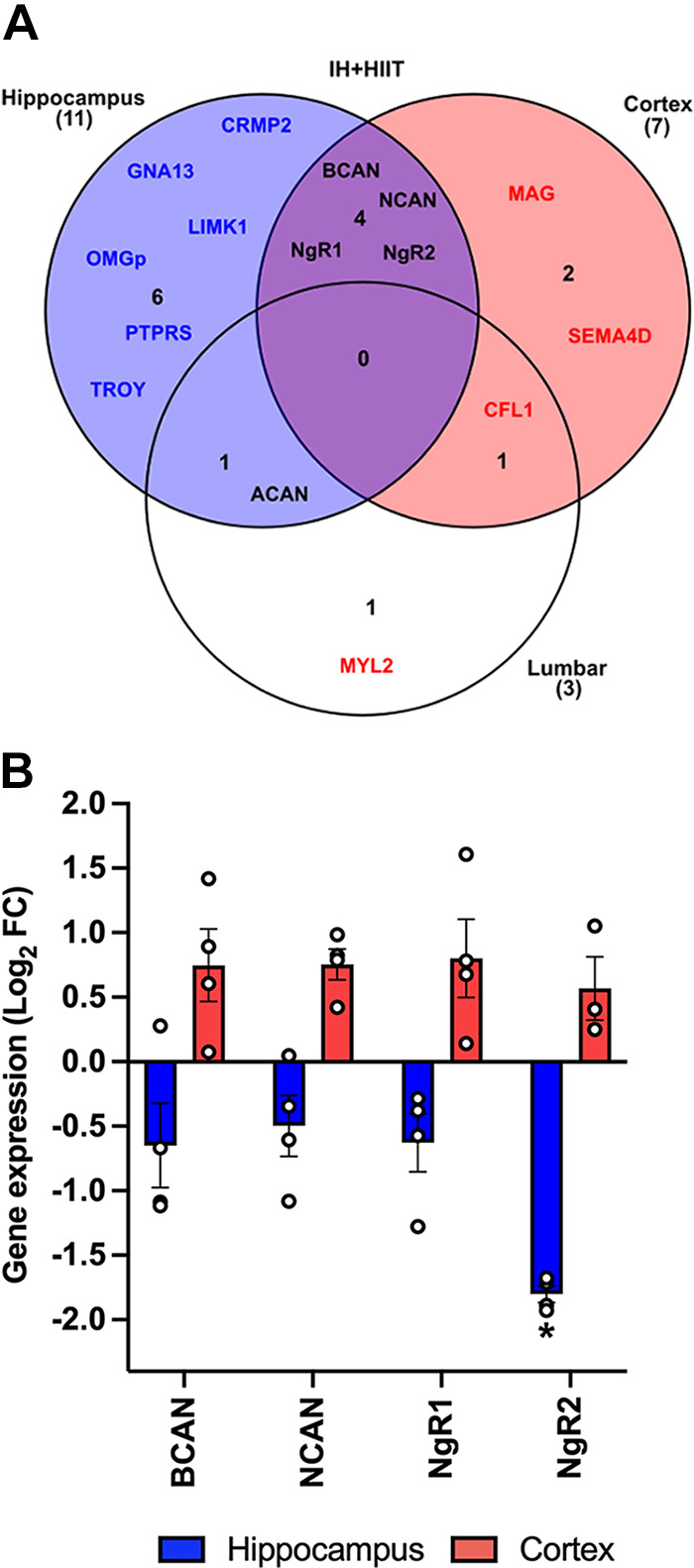
Differentially expressed RhoA/rho-associated coiled-coil containing protein kinase (ROCK) pathway genes following intermittent hypoxia (IH) with high-intensity interval training (HIIT) in the central nervous system (CNS). *A*: gene expression profiles were explored using the untransformed 2^−ΔΔCT^ cut-off values of ≤0.75 and ≥1.5 for relative fold change (FC) data to identify differentially expressed genes (DEGs) in the hippocampus, cortex, and lumbar regions after 6 wk of intermittent hypoxia combined with high-intensity interval training (IH+HIIT). Blue text—downregulated genes; red text—upregulated genes; black text—DEGs that have been identified in more than one CNS region with divergent expression patterns. *B*: the expression profiles of DEGs after IH + HIIT in the CNS. The relative fold change expression data for DEGs was Log_2_ transformed before statistical analysis. Blue bars—downregulated mRNA expression; red bars—upregulated mRNA expression. Depending on normality checks, differences in gene expression were assessed using one-way ANOVAs or Kruskal–Wallis *H* tests. Statistical significance determined by post hoc tests denoted with *P < 0.05 compared with sedentary control.

The IH + HIIT group showed similar differential gene expression patterns to IH within the CNS: in the hippocampus 20 of 21 genes (95%) had negative Log_2_ FC values; in the cortex 18 of 20 genes (90%) had positive Log_2_ FC values; and in the lumbar spinal cord 11 of 19 genes (58%) had positive Log_2_ FC values (Supplemental Tables S2, S3, and S4). Following IH + HIIT, there were no target genes identified as DEGs for all three CNS regions (Fig. 4*A*). However, four DEGs (*Bcan, Ncan, NgR1, and NgR2*) were downregulated in the hippocampus and upregulated in the cortex (Fig. 4*B*).

### RhoA/ROCK Pathway Transcriptional Changes following HIIT Were Mainly Observed in the Hippocampus

Following HIIT, there was no overlap of DEGs in hippocampal, cortical, and lumbar regions. In the hippocampus, HIIT downregulated seven DEGs (*Acan, Crmp2, Limk1, Ncan, NgR2, NgR3,* and *Ptprs*) and upregulated one DEG (*Mag).* However, gene expression profiles remained more stable following HIIT in the cortex and lumbar spinal cord. In the cortex, HIIT resulted in only one downregulated DEG, *Lpar1*. In the lumbar region of the spinal cord two DEGs were identified after HIIT: *Myl2* was upregulated and *Sema4D* was downregulated.

### Body Mass Gained throughout Training

The two groups including HIIT gained less body mass from the start to the end of the training protocols than the sedentary CON and IH groups. The percentage change of body mass in all experimental groups was not statistically significant compared with cage controls [CON 49.24 ± 7.15%; IH 54.80 ± 2.68%, *P* = 1.000; IH + HIIT 37.90 ± 4.08%, *P* = 0.072; HIIT 37.60 ± 6.53%, *P* = 0.062; *F*(3,12) = 9.930, *P* = 0.001]. Though, when compared with the IH group, the percentage of body mass gained was significantly lower in animals trained with HIIT (*P* = 0.004) and IH + HIIT (*P* = 0.005).

## DISCUSSION

Here, we provide evidence that the RhoA/ROCK signaling pathway can be regulated by a 6-wk training regime (30 min/day, 5 days a week) of IH, HIIT, or IH combined with HIIT. The overall gene profile of the RhoA/ROCK pathway was downregulated in the hippocampus following IH, IH + HIIT, and HIIT. Whereas, IH and IH + HIIT upregulated RhoA/ROCK genes in the cortex. The present results provide insight into potential further mechanisms at play in the regulation of structural neuroplasticity. To date, plasticity driven by interventions such as exercise and environmental enrichment is most prominently associated with the increase in neurotrophic factors such as BDNF (58–60). Here, we show that methods known for enhancing plasticity and upregulating neurotrophic factors (intermittent hypoxic training and exercise) can also modulate pathways responsible for restricting plasticity in the CNS.

It is important to note that changes in mRNA alone do not provide functional insight, and the functional outcome of the inhibitory molecules in this study will also depend on their phosphorylation status and protein levels. In addition, an increase or decrease in mRNA expression may be a compensatory response to previous decreased or increased pathway activity, respectively. However, this exploratory study provides an initial insight into which components of the RhoA/ROCK pathway are susceptible to change following IH, IH + HIIT, and HIIT.

The samples used for analysis in this study were tissue homogenates from hippocampal, cortical, and lumbar regions of the CNS and therefore we cannot discern what cell type the mRNA expression is changing within. The target genes studied within the RhoA/ROCK pathway included inhibitory ligands, membrane receptors, and downstream signaling molecules. The myelin-associated inhibitors Nogo-A, MAG, and OMGp are expressed by oligodendrocytes and neurons (29, 61). The guidance molecules EFNB3 and SEMA4D are also expressed by oligodendrocytes (30, 62). CSPGs (aggrecan, brevican, neurocan, and versican) are localized in the loose extracellular matrix and are found within PNNs surrounding GABAergic parvalbumin-positive fast-spiking interneurons throughout the CNS, excitatory pyramidal neurons in the CA2 region of the hippocampus, and motor neurons in the spinal cord (63–66). These inhibitory ligands bind to their respective membrane receptors upon the surface of neurons, and the activation of RhoA/ROCK and signaling through their downstream effectors occurs intracellularly (29, 67).

### Experimental Paradigms of IH and HIIT

The parameters of IH paradigms described in the literature vary in daily duration and study length of IH, the duration of hypoxic bouts and recovery intervals, and the level of hypoxia ($\text{F}{\text{I}}_{{\text{O}}_{2}}$) achieved. The use of chronic (7–8 h daily) and more severe doses of IH (4–10% $\text{F}{\text{I}}_{{\text{O}}_{2}}$) over study durations ranging from 10 days to 8 wk were shown to be detrimental within the CNS. Studies implementing these chronic/severe IH protocols in rodent models have reported impairments in neurogenesis (68, 69), spatial memory (70–72) and novel object recognition memory (73), reductions in the firing rate of hippocampal pyramidal neurons and dendritic spine density (74), downregulated expression of BDNF and synaptophysin (71, 72), suppression of long-term potentiation in the dentate gyrus (68), and an increase in oxidative stress (70). However, studies that expose rodents to IH for lower daily durations (4 h/day, ranging from 1 to 4 wk) and with less severe levels of hypoxia (11–16.3% $\text{F}{\text{I}}_{{\text{O}}_{2}}$) have been shown to enhance neuroplasticity and neuroprotection. The less severe protocols of IH were reported to enhance neurogenesis in wild-type rodent models (3–5). Furthermore, the less severe IH protocols were also documented to enhance hippocampal BDNF expression, and rescue impairments of neurogenesis and memory in models of strokes and Alzheimer’s pathology (6–9).

Similar to the less severe protocols of IH, HIIT (a form of exercise training that intersperses bouts of high-intensity exercise with bouts of rest or active recovery) has also been reported to enhance neural plasticity in rodents. As with IH, the parameters of HIIT paradigms are highly variable within the literature in terms of daily duration and study length of HIIT, the presence of a warm-up period, the duration and speeds ran on the treadmill during the high-intensity exercise bouts, and the duration and speed of rest or active recovery periods. However, HIIT studies with parameter ranges of: 4–8 wk study durations, 23–45 min daily exercise durations, 30 s–4 min high-intensity interval durations, and 1–3 min rest interval durations, were documented to enhance neurogenesis and spatial memory (10), and increase the hippocampal expression of BDNF, glial cell line derived neurotrophic factor (GDNF), and vascular endothelial growth factor (VEGF) (12–15).

As intermittent protocols of hypoxia and exercise training have both been associated with the enhanced expression of BDNF, this study aimed to investigate whether IH, HIIT, and implementing IH and HIIT in a combinatorial manner, could also modulate inhibitory molecules within the CNS. The IH paradigm used in the present study alternated five hypoxic intervals of 3 min at 15% $\text{F}{\text{I}}_{{\text{O}}_{2}}$, with five normoxic intervals of 3 min (total of 30 min/day, 5 days/wk, for 6 wk). In hypoxic intervals, oxygen was reduced to 15% $\text{F}{\text{I}}_{{\text{O}}_{2}}$ as this was within the range of administered hypoxia levels (11–16.3% $\text{F}{\text{I}}_{{\text{O}}_{2}}$) previously reported to induce neural plasticity.

Although the aforementioned literature supporting IH-induced neuroplasticity in the brain administered IH for 4 h/day, there is evidence that using shorter intervals of IH (5 min of 10.5–11% $\text{F}{\text{I}}_{{\text{O}}_{2}}$) induced phrenic motor plasticity, and improved breathing and forelimb function in models of spinal cord injury (46–48). As we wanted to administer IH and HIIT in a combinatorial manner, we also used shorter IH intervals (5 × 3 min) to align with a suitable HIIT protocol. The interval duration of 3 min allowed a steady state of hypoxia or normoxia to be achieved for roughly two and a half minutes. For the HIIT protocol, the interval durations (3 min each), the daily training duration (3-min warm up plus 30 min of interval training) and the total study duration (6 wk) were informed by the HIIT parameter ranges noted to enhance BDNF, neurogenesis, and improve spatial memory. To our knowledge, this is the first study that has combined IH with HIIT to investigate mechanisms underlying structural neuroplasticity. The IH and HIIT protocols were synchronized so that the animals would perform the higher intensity exercise bouts while being exposed to hypoxic conditions. It is possible that the increased demand for oxygen during HIIT coupled with the reduced oxygen availability from IH may result in greater hyperventilation and hypocapnia (75), thus creating an additional variable when using these protocols in tandem. This should be taken into consideration for future research investigating the mechanisms of how IH, HIIT, and IH + HIIT regulate the expression of inhibitory molecules.

Throughout this study, animals did not display any visual signs of an adverse response to the experimental protocols e.g., grimace, pain, abnormal respiration, grooming, motor posture, or appetite (76, 77). The body mass gained throughout the study was lower in animals that completed exercise training compared with the sedentary control and IH groups, which is biologically plausible as they had higher energy expenditure. Though, body mass changes in all experimental groups did not statistically differ compared with cage controls.

### IH, IH ± HIIT, and HIIT Downregulated Inhibitory Molecules in the Hippocampus

In the hippocampus, 6 wk of either IH or HIIT alone significantly downregulated the mRNA expression of *Acan* (aggrecan). The CSPG aggrecan is a main component of PNNs that are aggregated extracellular matrix structures that form around certain neuronal subtypes in the CNS, and are known to restrict plasticity (78). Although the present study is the first to show that CSPG gene expression can be regulated by IH, it is not the first to show that exercise may also modulate CSPGs; 6 wk of ad libitum voluntary wheel running has previously been shown to reduce the number and thickness of PNNs in the hippocampus (45). In addition, PNNs were reduced in the deep cerebellar nuclei during the acquisition of a motor learning task (eye blink conditioning) (79). Together, these results support that PNNs are involved in, or are susceptible to, modulation from experience-driven plasticity. PNNs in the brain have also been the target of experimental manipulation to functionally improve cognition. The enzymatic removal of PNNs in the perirhinal cortex using chondroitinase ABC improved novel object recognition memory (80). Similarly, the removal of PNNs using a brain-wide genetic knockout of *Acan* also enhanced novel object recognition memory (81). The downregulation of *Acan* expression in the present study suggests that *Acan*, and therefore PNNs, may be modulated in the hippocampus in response to IH and HIIT. As the hippocampus is a brain region associated with learning and memory it is possible that, like in other brain regions, modulation of hippocampal PNNs may also facilitate memory improvements. However, the role of hippocampal PNNs in memory is yet to be ascertained.

In addition to *Acan*, IH, IH + HIIT, and HIIT downregulated hippocampal *NgR2* mRNA expression. NgR2 is a receptor for the myelin-associated inhibitor, MAG (82), and has been shown to restrict hippocampal dendritic spine formation (83). Furthermore, the knockdown of *NgR2* was reported to induce a shift toward a higher proportion of mushroom-shaped dendritic spines (84). As dendritic spine formation, and the mushroom morphology of dendritic spines, are thought to underlie memory formation (85), it is possible that the downregulation of hippocampal *NgR2* mRNA expression in the present study may also facilitate memory improvements. IH has previously been shown to alleviate memory impairments induced by excitotoxic brain lesions in postnatal mice (86), focal cerebral ischemia (7, 8), and in models of Alzheimer’s disease (6). This further supports that the hippocampal candidates identified in the present study (aggrecan and nogo-receptor 2) may play a role in IH-induced memory improvements.

### IH and IH ± HIIT Upregulated Inhibitory Molecules in the Cortex

In contrast to the downregulation of RhoA/ROCK pathway components in the hippocampus, IH significantly upregulated the mRNA expression of *Lingo1* and *NgR3* in the cortex. NgR3, a known CSPG receptor (56), is important for axon pathfinding in the embryonic spinal cord (87), and restricts the formation of hippocampal dendritic spines (83). However, the functional role of NgR3 specifically in cortical regions is yet to be elucidated.

LINGO-1, a transmembrane coreceptor of NgR1, is known to negatively regulate oligodendrocyte differentiation and myelination, and is expressed on oligodendrocytes and neurons (88). Within the NgR1/LINGO-1/TROY receptor complex, LINGO-1 transfers the extracellular signal to the cytosolic environment and triggers the intracellular signaling cascade (RhoA/ROCK pathway) following the binding of Nogo-A, MAG, or OMGp, to NgR1 (89). Elevated LINGO-1 expression is associated with neural injury and several CNS diseases including spinal cord injury, ischemic strokes, Alzheimer’s disease, Parkinson’s disease, and schizophrenia (89–93). Similar to the current study, the continuous exposure to hypoxia (10% O_2_) for 2 wk was also shown to upregulate *Lingo1* expression and increased depression-like behavior (94). In addition, the hypoxia-induced depression was attenuated by blocking RhoA/ROCK signaling (94). Thus, the upregulation of *Lingo1* in the present study may be reflective of a deleterious environment in cortical regions following IH.

### Divergent Response of the Hippocampus and Cortex to the Same IH Stimulus

The previously discussed literature suggests that the present IH protocols may enhance neuroplasticity within the hippocampus yet create an inhibitory environment within the cerebral cortex. We are the first to investigate whether IH, IH + HIIT, and HIIT could regulate the expression of inhibitory molecules with the CNS, and to our knowledge this is the first time training protocols of this nature have been used within a neuroscience context. Our hippocampal data showing downregulation of *Acan* following IH and HIIT is in accordance with literature showing a downregulation of CSPGs following 6 wk of voluntary wheel running (45). We also hypothesized there would be downregulation of inhibitory molecules in the cortex as 1 wk of voluntary wheel running downregulated Nogo-A expression in cortical tissue (43). In addition, the reduction in hippocampal Nogo-A levels was correlated with the distance ran in a voluntary wheel running study (42). Voluntary wheel running paradigms involve much higher volumes of exercise than our HIIT protocol, therefore higher volumes of exercise may be required to downregulate inhibitory molecules in the cortex. We did however see an upregulation of inhibitory molecules in the cortex in response to IH. Research investigating the effect of IH on the expression of inhibitory molecules is lacking, however, there is some evidence to show that the hippocampus and the cortex respond differently to the same hypoxic stimulus. In a rodent model of Alzheimer’s disease, 2 wk of intermittent hypoxia (14.3% O_2_, 4 h/day) reduced the number of β-amyloid plaques in the hippocampus yet not in the cortex (9). In anesthetized rats, the partial pressure of oxygen was higher and the levels of the hypoxia marker, pimonidazole, were lower in the dentate gyrus region of the hippocampus compared with the cerebral cortex during normoxic conditions (95). This suggests there is differential oxidative metabolism between hippocampal and cortical regions, which may support why these brain regions responded differently to the hypoxic stimulus in this study. To characterize the neuroplastic effects that are associated with the differential regulation of RhoA/ROCK pathway, further research into the structural changes that occur in the hippocampus and cortex following IH is warranted.

### Conclusions and Future Impact

Our results demonstrate that genes within the RhoA/ROCK signaling pathway can be regulated by IH, IH + HIIT, and HIIT in the brain. In the hippocampus, IH and HIIT downregulated *Acan,* and IH, IH + HIIT, and HIIT downregulated *NgR2*; in the cortex IH and IH + HIIT upregulated *Lingo1*, and IH alone upregulated *NgR3*. The functional relevance of these transcriptional changes requires further investigation. However, we have shown that inhibitory pathways are also regulated by interventions previously known to upregulate neurotrophic factors. This provides a mechanistic insight that will potentially lead to a more holistic understanding of how neuroplasticity can be manipulated in the mature CNS. The genetic knockdown of the candidates downregulated in the hippocampus following IH, HIIT, and IH + HIIT, *Acan* and *NgR2*, has previously been associated with improved memory, and the increase in hippocampal dendritic spine formation, respectively. The hypoxia- or HIIT-induced downregulation of *Acan* and *NgR2* may facilitate improvements in learning and memory and suggests a mechanistic link between these interventions and the alleviation of cognitive decline. HIIT is an affordable and accessible form of exercise training that can be performed at home. Furthermore, IH is an intervention suitable for a wide range of patient populations, especially where capacity for movement is low, as it can be easily administered through a facemask that provides oxygen delivery at a set concentration. Though, to determine the therapeutic potential of IH for cognitive decline, it needs to be established whether the upregulation of RhoA/ROCK pathway genes in the cortex acts to prevent maladaptive plasticity or induces a disease-like state. Future research should investigate how IH and HIIT affect the protein levels of the highlighted candidates, how the candidates interact with neurotrophic factors (e.g., BDNF), the structural changes associated with expression changes (e.g., whether their levels are associated with the outgrowth of dendritic trees from adult-born granule cells or morphological differences in dendritic spines), and whether this leads to functional changes e.g., in learning and memory. These results could highlight targets of structural neuroplasticity that can be modulated through easy-to-access treatments for a wide variety of conditions in the future.

## DATA AVAILABILITY

Data will be made available upon reasonable request.

## SUPPLEMENTAL MATERIAL

10.17632/tf4khphz53.1 Supplemental Tables S1–S5: https://doi.org/10.17632/tf4khphz53.1.

## GRANTS

This research was funded through a University of Leeds PhD scholarship awarded to Natalie E. Doody. Nicole J. Smith was funded by The Medical Research Council Discovery Medicine North Doctoral Training Programme (MRC DiMeN DTP) under Grant Number 95505174.

## DISCLOSURES

No conflicts of interest, financial or otherwise, are declared by the authors.

## AUTHOR CONTRIBUTIONS

N.E.D., G.N.A., J.C.F.K., and R.M.I. conceived and designed research; N.E.D., N.J.S., and E.C.A. performed experiments; N.E.D. and E.C.A. analyzed data; N.E.D., E.C.A., G.N.A., J.C.F.K., and R.M.I. interpreted results of experiments; N.E.D. and N.J.S. prepared figures; N.E.D. drafted manuscript; N.E.D., N.J.S., E.C.A., G.N.A., J.C.F.K., and R.M.I. edited and revised manuscript; N.E.D., N.J.S., E.C.A., G.N.A., J.C.F.K., and R.M.I. approved final version of manuscript.
